# Insights into the Genetic Diversity of *Yersinia enterocolitica* Isolated from Poultry and Red Meat in South Korea in 2024

**DOI:** 10.3390/pathogens15080821

**Published:** 2026-08-04

**Authors:** Dabin Kim, Sumin Ryu, Yeeun Kim, Jaehyun Choi, Min Jung Lee, Yonghoon Kim, Insun Joo, Woojung Lee

**Affiliations:** Division of Food Microbiology, National Institute of Food and Drug Safety Evaluation, Ministry of Food and Drug Safety, Cheongju 28159, Republic of Korea; dabin29@korea.kr (D.K.); banasm@korea.kr (S.R.); kye76@korea.kr (Y.K.); wogus58210@korea.kr (J.C.); ymin0110@korea.kr (M.J.L.); washout71@korea.kr (Y.K.); jis901@korea.kr (I.J.)

**Keywords:** *Yersinia enterocolitica*, comparative genomics, whole-genome sequencing, genome surveillance, food safety

## Abstract

*Yersinia enterocolitica* is a psychrotrophic foodborne bacterium that can proliferate at refrigeration temperatures and is frequently associated with animal-source foods, raising concerns about food safety and public health. However, genomic data on *Y. enterocolitica* isolates from South Korea are limited, despite the increasing use of whole-genome sequencing (WGS) in bacterial surveillance. Herein, 91 *Y. enterocolitica* isolates recovered from chicken, pork, beef, and duck samples collected nationwide in 2024 were analyzed using WGS to elucidate their genomic diversity and genomic features. Phylogenomic analysis predicted all isolates as biotype 1A (sub-biotype 1Aa) and revealed substantial genetic diversity, comprising 27 sequence types and 43 core-genome types. Pan-genome analysis identified 11,230 gene clusters, revealing an open pan-genome in which accessory and unique gene clusters were assigned to predicted functional categories associated with metabolism, defense mechanisms, and stress responses. Although the canonical virulence plasmid pYV was absent, conserved chromosomal virulence-associated genes involved in adhesion (*yapE*), invasion (*inv*), secretion, and enterotoxicity (*ystB*) were detected. Antimicrobial resistance genes were predominantly intrinsic, particularly *blaA* and *vat(F)*, whereas acquired resistance genes were identified sporadically. These findings provide a genomic baseline for biotype 1A *Y. enterocolitica* isolates recovered from animal-source foods in South Korea. The functional and public health significance of the detected virulence-associated loci requires further phenotypic investigation.

## 1. Introduction

*Yersinia enterocolitica* is a psychrotrophic zoonotic bacterium responsible for foodborne yersiniosis, a gastrointestinal infection characterized by diarrhea, fever, and mesenteric lymphadenitis, and, in some cases, associated with systemic complications such as sepsis and reactive arthritis [1,2]. In the European Union, yersiniosis consistently ranks among the top three reported zoonoses, with most cases attributed to *Y. enterocolitica* [3]. Transmission typically occurs through the consumption of contaminated animal-source foods, particularly pork, which is recognized as the primary reservoir for pathogenic *Y. enterocolitica* [4,5]. Contamination has also been reported in other animal-derived foods, including beef, chicken, and duck, reflecting the persistence of this pathogen in slaughter and processing environments [6,7]. The organism’s ability to survive and proliferate at refrigeration temperatures underscores its significance in food safety and highlights the need for continuous monitoring across livestock production and cold-chain systems [8,9,10].

Traditionally, *Y. enterocolitica* has been classified into six biotypes (1A, 1B, and 2–5) based on its pathogenic potential [11]. Biotype 1B is highly virulent and responsible for most human yersiniosis cases, whereas biotypes 2–5 display moderate pathogenicity [12]. Biotype 1A has long been regarded as nonpathogenic, as it typically lacks the virulence plasmid (pYV) and key chromosomal markers such as *ail* and *ystA* [13]. However, genomic and epidemiological evidence indicates that biotype 1A is genetically diverse and may harbor loci associated with adhesion or enterotoxicity, including *ystB*, *inv*, and *yaxA*/*B* [14,15]. These findings suggest that some 1A lineages adapt to environmental niches and exhibit limited pathogenic potential under specific conditions [16].

Beyond the traditional biotyping framework, advances in whole-genome sequencing (WGS) have enabled high-resolution characterization of *Y. enterocolitica*, including biotype prediction, sequence typing, and in silico identification of virulence factors (VFs), antimicrobial resistance (AMR) genes, and plasmid-associated elements [17,18,19]. WGS-based approaches have increasingly been applied to population structure analysis, outbreak investigations, and evolutionary studies of *Y. enterocolitica* in multiple countries [20,21,22]. Such genomic data provide critical insights into the population structure, virulence potential, and AMR profiles of strains circulating in food-associated environments. However, despite advanced livestock production and distribution systems in South Korea, comprehensive genome-based studies of *Y. enterocolitica* from animal-source foods remain limited.

Herein, a comprehensive genomic analysis was conducted on *Y. enterocolitica* isolates recovered from animal-source foods, including chicken, pork, beef, and duck, across South Korea in 2024. By integrating WGS-based comparative and phylogenomic analyses, the genetic diversity, population structure, and genomic characteristics of the isolates were assessed. This study provides baseline genomic information on food-derived *Y. enterocolitica* in South Korea and represents the first WGS-based characterization of isolates recovered from animal-source foods in the country.

## 2. Materials and Methods

### 2.1. Bacterial Strains

*Y. enterocolitica* strains (n = 91) were obtained in 2024 from animal-source foods, including beef, pork, chicken, and duck, in South Korea and were provided by the Korean Culture Collection for Foodborne Pathogens (Ministry of Food and Drug Safety, Cheongju, Republic of Korea). An overview of the isolates is presented in Table 1, and detailed metadata for each isolate are provided in Supplementary Table S1. Strain identification was performed using a VITEK MS system (BioMérieux Inc., Marcy-l’Étoile, France).

### 2.2. WGS

Genomic DNA was extracted from 91 isolates using the Qiagen DNeasy Blood & Tissue Kit (Qiagen, Hilden, Germany) according to the manufacturer’s instructions. DNA concentration was measured using a Qubit 4 Fluorometer (Thermo Fisher Scientific, Waltham, MA, USA).

Sequencing libraries were prepared using the Illumina DNA Prep Kit (Illumina, San Diego, CA, USA) and sequenced on an Illumina NextSeq 1000/2000 platform, generating 300-bp paired-end reads. The resulting reads were assembled de novo using default k-mer settings in CLC Genomics Workbench v12.0 (Qiagen). Assembly statistics, including genome size, number of contigs, and N50, were calculated using QUAST v5.2.0. Genome completeness and contamination were assessed using CheckM2 v1.1.0. Genomes with estimated completeness of ≥95% and contamination of ≤10% were retained for downstream analyses. Isolate-level sequencing depth and genome assembly quality metrics are provided in Supplementary Table S2.

### 2.3. In Silico Genotyping

In silico genotyping was performed for multilocus sequence typing (MLST) and core-genome MLST (cgMLST). MLST and cgMLST profiles of *Y. enterocolitica* were assigned using the EnteroBase platform (https://enterobase.warwick.ac.uk, accessed on 23 April 2026). The MLST scheme followed the Achtman 7-gene scheme (*adk*, *argA*, *aroA*, *glnA*, *thrA*, *tmk*, and *trpE*). cgMLST allele profiles were based on 1553 loci defined in the EnteroBase Yersinia cgMLST scheme, representing a soft-core genome composed of loci present in at least 95% of reference genomes. Pairwise allelic distances were calculated using cgmlst-dists (https://github.com/tseemann/cgmlst-dists, accessed on 20 May 2026). The number of successfully assigned cgMLST loci was determined for each isolate from the 1553-locus cgMLST profile.

### 2.4. Pan-Genome Analysis and COG Functional Classification

Pan-genome analysis was performed using the Bacterial Pan Genome Analysis pipeline (BPGA v1.3) with default parameters. Predicted protein sequences were clustered into orthologous gene clusters using USEARCH, the default clustering algorithm implemented in the pipeline, with an amino acid sequence identity cutoff of 50%. The pan-genome profile of the *Y. enterocolitica* isolates was characterized based on the numbers of core, accessory, and unique gene clusters. Gene clusters present in all 91 genomes were defined as core gene clusters, those present in 2–90 genomes as accessory gene clusters, and those present in only one genome as unique gene clusters. Functional classification of the core, accessory, and unique gene clusters was performed according to Clusters of Orthologous Groups (COG) categories using the functional analysis module implemented in the pipeline with default settings.

### 2.5. Biotype Prediction

For biotype prediction, 57 publicly available *Y. enterocolitica* genomes with known biotypes and sub-biotypes were retrieved from previous studies [18] (Table S3) and analyzed alongside the 91 study isolates within the pan-genome framework. A pan-genome-based neighbor-joining phylogenetic tree was constructed from a binary presence/absence matrix of orthologous gene clusters. Isolates were assigned to biotypes and sub-biotypes based on their clustering with the corresponding reference genomes. The resulting tree was visualized using iTOL v6.

### 2.6. Genomic Features of Y. enterocolitica

Genomic features of *Y. enterocolitica* were characterized with a focus on VFs, AMR genes, and plasmid replicons. These features were identified using ABRicate v1.0.1 (https://github.com/tseemann/abricate, accessed on 23 April 2026) with default BLAST-based filtering parameters, applying a minimum nucleotide identity threshold of 80% and a minimum coverage threshold of 80%. Screening was performed against three databases: Virulence Factor Database, ResFinder, and PlasmidFinder. For AMR genes, additional confirmation of gene family assignment and predicted resistance phenotype was based on ResFinder annotations. PlasmidFinder hits obtained from short-read assemblies were interpreted as plasmid replicon detections rather than confirmation of complete or circular plasmids.

## 3. Results

### 3.1. Isolates

A total of 91 *Y. enterocolitica* strains isolated in 2024 from animal-source foods—including chicken (32/91), pork (30/91), beef (24/91), and duck (5/91)—were included in this study. The isolates were collected across different regions of South Korea (Table 1; Table S1). They represented a broad geographic distribution, with samples originating from Gyeonggi (7/91), Gangwon (1/91), Chungcheong (22/91), Jeolla (52/91), and Gyeongsang (9/91) provinces. All genomes showed high estimated completeness, ranging from 99.58% to 100.00% (median, 100.00%), while estimated contamination ranged from 0.00% to 7.43% (median, 0.04%) (Table S2).

A neighbor-joining phylogenetic tree was constructed based on a binary presence/absence matrix of orthologous gene clusters from 91 *Y. enterocolitica* isolates collected in 2024 in South Korea, together with reference genomes representing known biotypes (1A, 1B, 2, 3, 4, and 5) and sub-biotypes (1Aa and 1Ab). Colored outer rings indicate predicted biotype and sub-biotype classifications, while reference genomes are marked with navy circles. The phylogenetic tree was visualized using iTOL v6.

### 3.2. Biotyping

**Figure 1:**
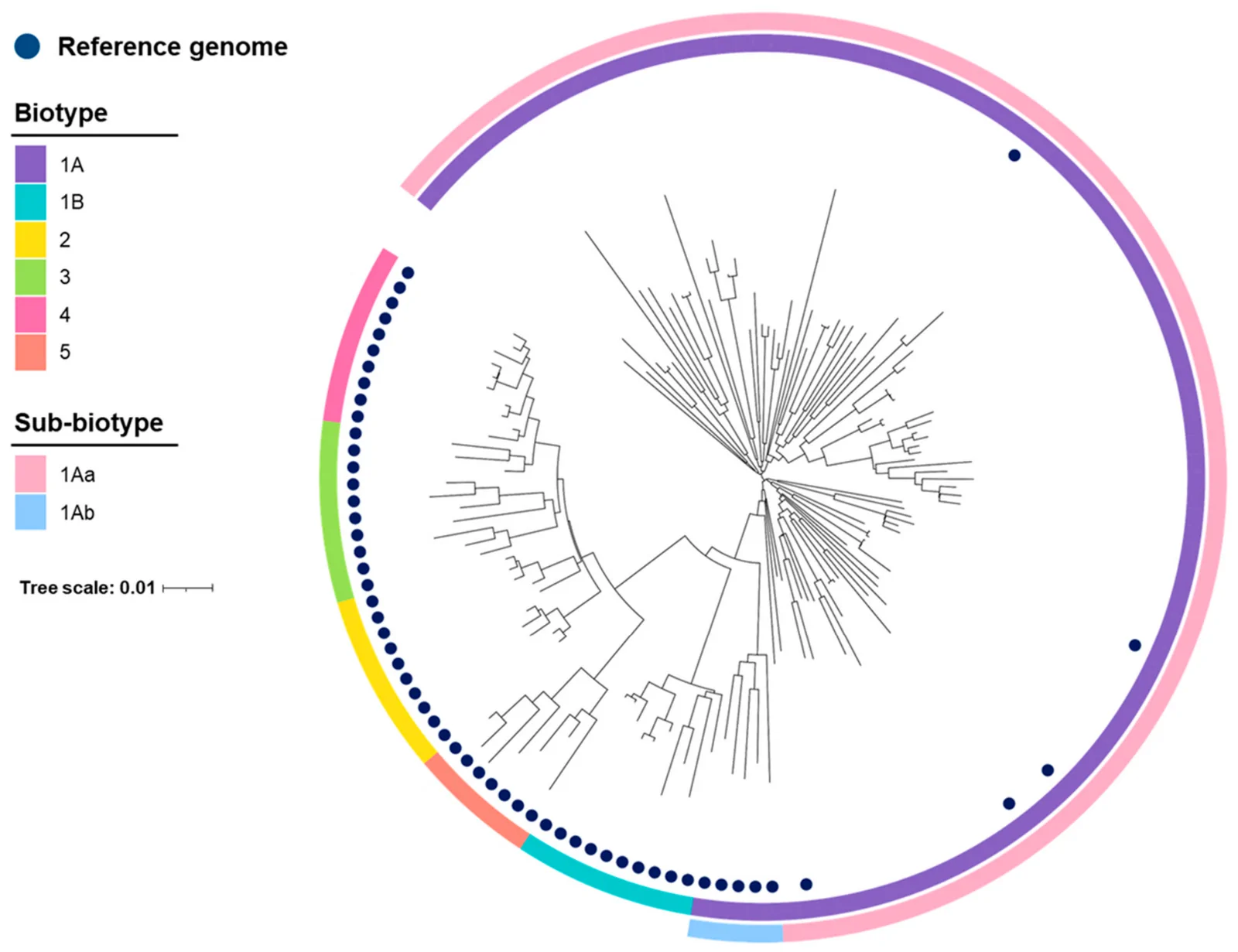
Phylogenetic relationships of *Y. enterocolitica* isolates based on a binary presence/absence matrix of orthologous gene clusters.

Phylogenetic analysis based on a binary presence/absence matrix of orthologous gene clusters was conducted to predict the biotypes of the 91 isolates (Figure 1). In the resulting phylogeny, reference strains of each biotype consistently clustered together, supporting the reliability of the biotype predictions. All isolates were genomically predicted as biotype 1A (100%). To further resolve intra-biotype variation, the 91 isolates were compared with reference genomes representing the sub-biotypes 1Aa and 1Ab. These isolates clustered with the 1Aa reference genomes, indicating that all isolates analyzed in this study were genomically consistent with sub-biotype 1Aa.

### 3.3. Sequence Type (ST) Diversity

The *Y. enterocolitica* isolates recovered from animal-source foods were assigned to 27 distinct STs and 43 core-genome types (CTs), while six isolates remained unassigned because their complete allelic profiles did not correspond to any ST currently registered in the MLST database (Table 1; Table S1). Among these, ST134 was predominant, accounting for 23 of the 91 isolates (25.3%), followed by ST179 (12 isolates, 13.1%), ST150 (6 isolates, 6.6%), and ST246 (6 isolates, 6.6%). Overall, MLST indicated a genetically diverse population structure characterized by a few dominant STs and numerous rare types, consistent with ongoing diversification within *Y. enterocolitica* populations associated with animal-source foods in South Korea. Within the predominant ST134 lineage, multiple distinct CTs were identified, indicating substantial genomic heterogeneity. Specifically, ST134 isolates from chicken were assigned to CT1420 and CT3053; those from pork to CT480, CT1548, and CT3053; those from duck to CT480 and CT3053; and those from beef to CT3053. Notably, CT3053 was identified among ST134 isolates from all four food categories.

**Figure 2:**
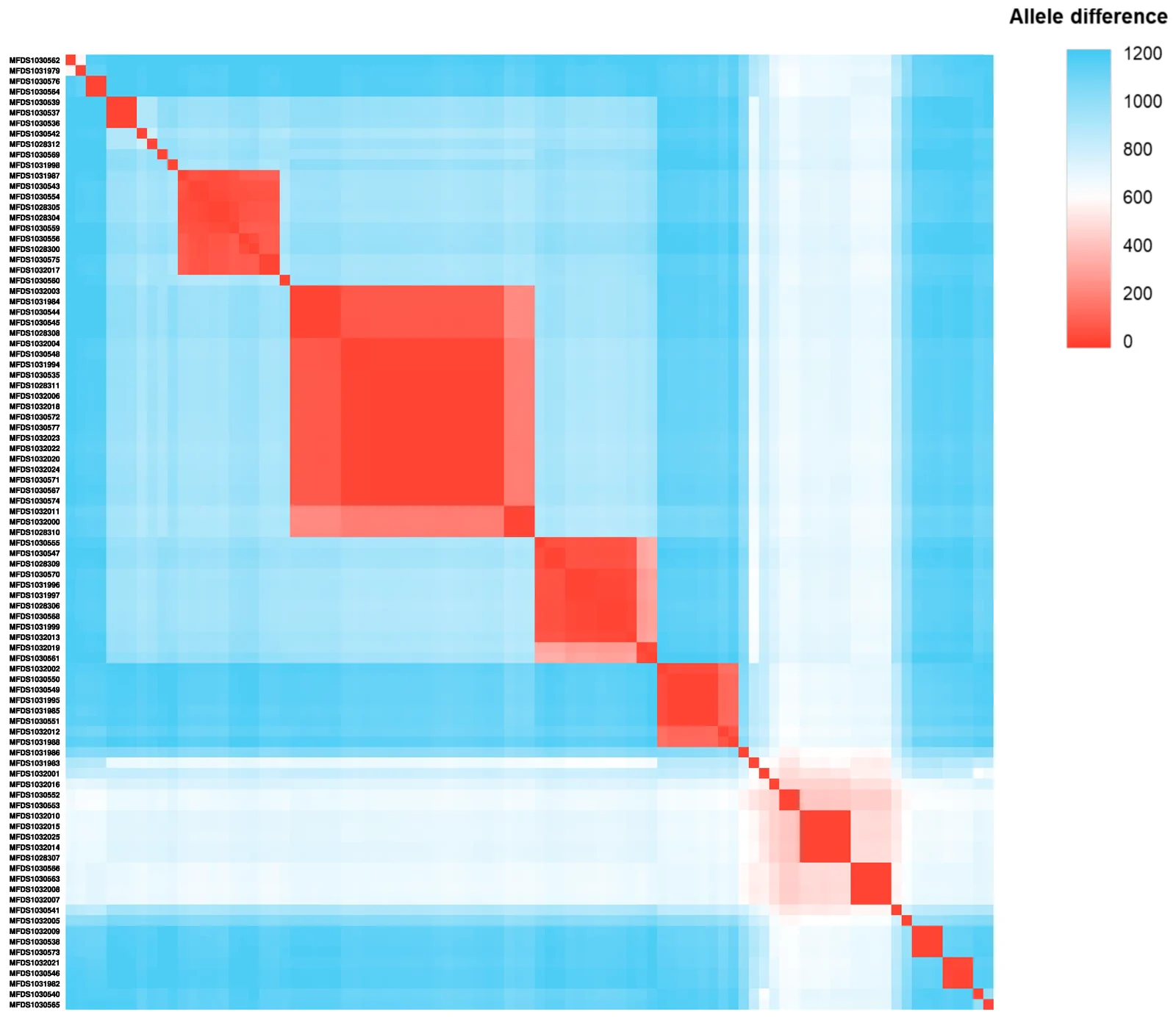
Allelic distance heatmap of *Y. enterocolitica* isolates based on core-genome multilocus sequence typing (cgMLST). Pairwise allelic distances among 91 *Y. enterocolitica* isolates were calculated using the EnteroBase cgMLST scheme (1553 loci). The heatmap depicts genomic relatedness between isolates, with red indicating high similarity (low allelic distance) and blue indicating greater divergence.

Allelic relationships among isolates were visualized using a cgMLST allele-distance heatmap (Figure 2). Across the 4095 pairwise isolate comparisons, cgMLST allelic distances ranged from 0 to 1273, with a median pairwise distance of 934 (IQR, 684–1135). A total of 61 isolate pairs showed zero allelic differences across shared assigned loci, forming 18 zero-distance groups involving 52 isolates. Separately, the number of successfully assigned cgMLST loci per isolate ranged from 845 to 1473 of the 1553 loci, with a median of 1423 loci. Isolate-level assigned-locus counts and zero-distance group designations are provided in Supplementary Table S4.

### 3.4. Pan-Genome and COG Analysis

**Figure 3:**
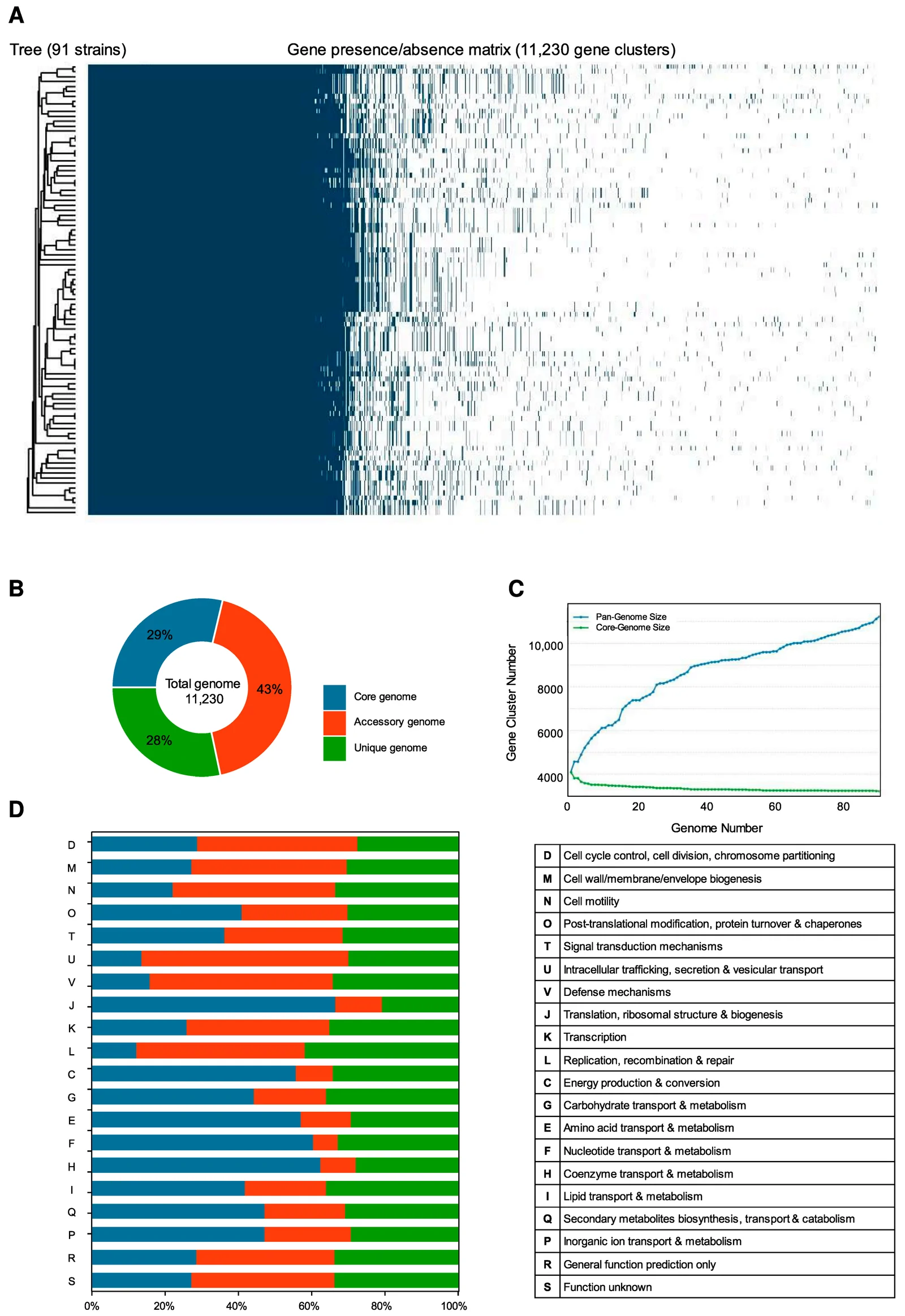
Pan-genome analysis and functional classification of *Y. enterocolitica* isolates collected in South Korea. (**A**) Gene presence/absence matrix of 11,230 gene clusters among 91 isolates, aligned with a hierarchical tree depicting their genetic relationships. (**B**) Proportional composition of core (29%), accessory (43%), and unique (28%) genes within the pan-genome. (**C**) Pan- and core-genome accumulation curves illustrating the expansion of total gene clusters and the plateauing of core genes with the addition of new genomes. (**D**) Functional classification of core, accessory, and unique genes based on Clusters of Orthologous Groups (COG) categories.

Pan-genome analysis identified 11,230 gene clusters among the isolates, comprising 3224 core (29%), 4828 accessory (43%), and 3178 unique (28%) genes (Figure 3A–C). Each isolate exhibited a distinct gene presence/absence profile, and pan- and core-genome accumulation curves indicated a continuously expanding pan-genome with a stable core genome, consistent with an open pan-genome structure.

COG-based functional classification showed that accessory and unique gene clusters were assigned to a broad range of predicted functional categories, including metabolism, cellular processes and signaling, and information storage and processing (Figure 3D; Table S5). In particular, these variable gene clusters were assigned to categories related to metabolism (e.g., carbohydrate, amino acid, and lipid transport and metabolism), cellular processes (e.g., signal transduction and defense mechanisms), and information storage and processing (e.g., replication, recombination, and repair). In contrast, core genes were more strongly represented in categories associated with essential cellular functions, such as translation, energy production and conversion, and nucleotide transport and metabolism.

Overall, these findings indicate that the isolates share a conserved genomic backbone while differing in their accessory gene content, reflecting underlying genomic diversity.

### 3.5. Genomic Features: VFs, AMR Genes, and Plasmid Replicons

#### 3.5.1. VFs

**Figure 4:**
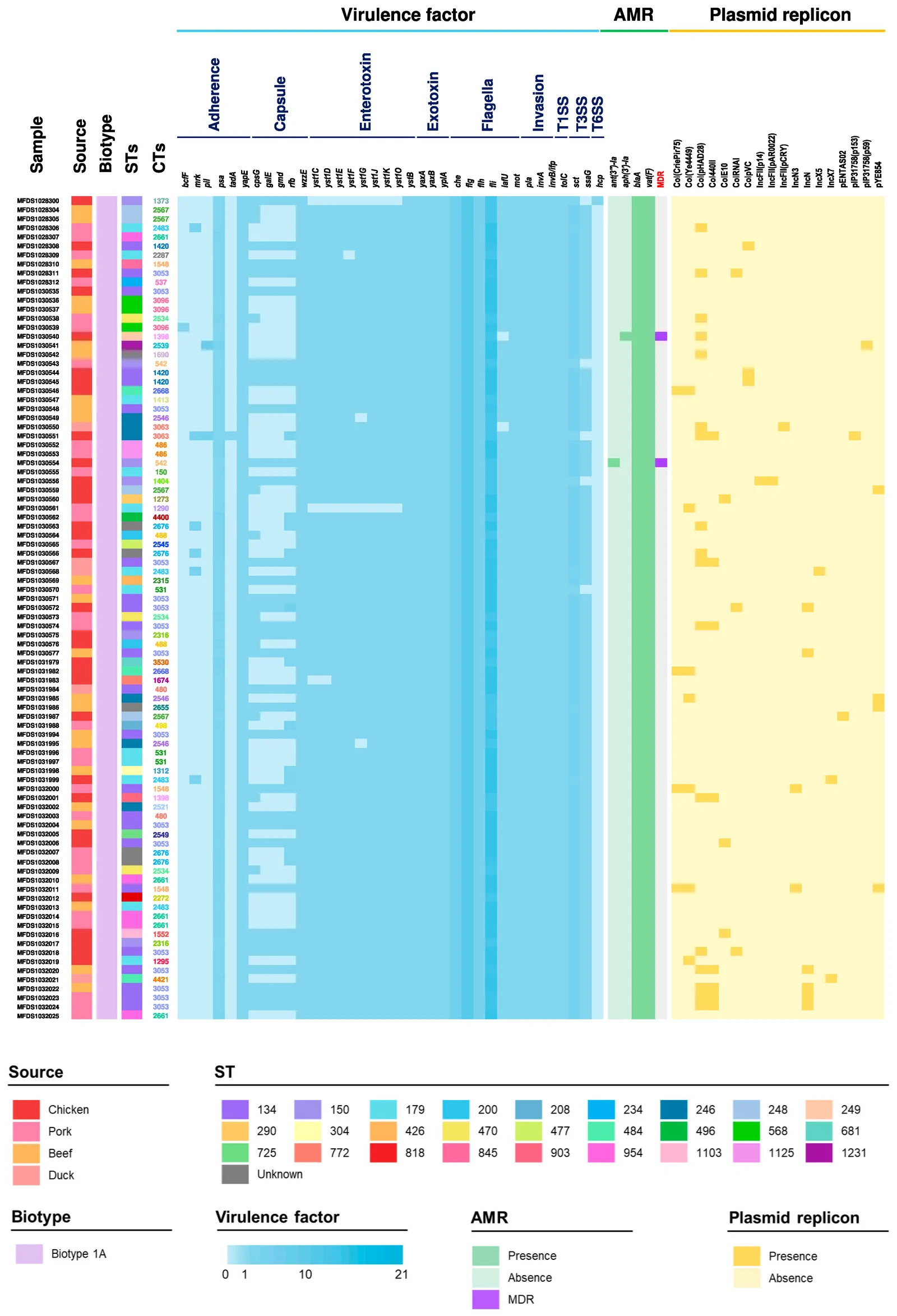
Distribution of virulence factors (VFs), antimicrobial resistance (AMR) genes, and plasmid replicons among *Y. enterocolitica* isolates. Each row represents an isolate, and each column represents a genomic feature. Metadata columns on the left indicate isolation source, biotype, sequence type (ST), and core-genome type (CT). VF-associated genes are shown in blue and grouped by functional category, including adherence, capsule formation, enterotoxin, exotoxin, flagella, invasion, and secretion systems (type I secretion system [T1SS], type III secretion system [T3SS], and type VI secretion system [T6SS]). AMR genes are shown in green, while multidrug resistance (MDR) is indicated in red font. Plasmid replicons are shown in yellow. Colored cells indicate the presence of each gene or replicon, whereas blank cells indicate its absence.

Screening of the 91 *Y. enterocolitica* isolates identified 107 virulence-associated genes grouped into 9 functional categories, including adherence, capsule formation, enterotoxin, exotoxin, flagella, invasion, and secretion systems (type I secretion system [T1SS], type III secretion system [T3SS], and type VI secretion system [T6SS]) (Figure 4; Table S6). Many virulence-associated genes, particularly those related to enterotoxin production, cytotoxicity/proteolytic activity, invasion, motility, and secretion systems, were widely distributed among the isolates, whereas adherence- and capsule-associated genes showed greater variability.

All isolates carried several adhesion-related determinants. The *psa* locus showed variable distribution: *psaC* was detected in all isolates, whereas *psaE* and *psaF* were detected in 30.8% and 83.5% of isolates, respectively. In contrast, *psaA* and *psaB* were identified only sporadically (1.1% and 4.4%, respectively). Additionally, the autotransporter gene *yapE* was present in all isolates. Other adherence-related genes, including *bcfF*, *mrk* cluster genes, type IV pilus-associated genes, and *tadA*, were detected at low frequencies, ranging from 1.1% to 6.6%. Capsule-associated genes also showed variable distributions. Although *wzzE* was present in all isolates, other capsule- or O-antigen–related genes, including *cpsG*, *galE*, *gmd*, and *rfb* cluster genes, exhibited variable carriage rates ranging from 3.3% to 47.3%. Overall, adherence- and capsule-related genes exhibited greater variability among isolates, whereas enterotoxin-associated genes were largely conserved. *ystB* was detected in all isolates, and genes encoding secretion-related components, including *yst1C*, *yst1D*, *yst1E*, *yst1F*, *yst1G*, *yst1J*, *yst1K*, and *yst1O*, were detected in more than 95% of isolates. Exotoxin-associated genes, *yaxA*, *yaxB*, and *yplA*, were present in all isolates. The invasion-associated genes *pla*, *invA*, and *invB/ifp* were also detected in all isolates, indicating that these loci are highly conserved in the examined isolates. Flagellar and chemotaxis-associated genes were widely distributed, with most detected in all isolates, indicating that motility-associated functions were largely conserved. However, some flagellar genes, such as *fliB*, *fliC*, and *fliD*, showed lower carriage rates (58.2–67.0%), indicating limited variation within this functional category. All isolates contained *tolC*, which encodes the outer-membrane channel of the T1SS. Multiple T3SS genes, *sctD*, *sctI*, *sctJ*, *sctL*, and *sctR*, were detected in 94.5–98.9% of isolates, while the SPI-2–like *ssaG* gene was present in 94.5% of isolates. In contrast, the T6SS-associated gene *hcp2* was detected in only one isolate.

Overall, toxin-, invasion-, motility-, and secretion-associated genes were generally conserved among the isolates, while adherence- and capsule-associated genes showed greater variability. These findings indicate that *Y. enterocolitica* isolates from animal-source foods possess a broadly conserved set of virulence-associated genes, while selected surface-associated determinants vary among isolates. This pattern may reflect a stable genomic background with isolate-level differences in traits related to adhesion, capsule formation, and host or environmental interactions.

#### 3.5.2. AMR Genes

All isolates carried the chromosomally encoded *blaA* (intrinsic β-lactam resistance) and *vat(F)* (streptogramin inactivation) genes, indicating a conserved baseline resistance profile (Figure 4; Table S7). Two isolates were classified as multidrug-resistant (MDR), each carrying acquired AMR genes conferring resistance to additional antimicrobial classes. Specifically, the isolate MFDS1030540 carried *aph(3′)-Ia*, while MFDS1030554 carried *ant(3″)-Ia*. Overall, the detected AMR genes were predominantly associated with enzymatic antibiotic inactivation, including β-lactam inactivation mediated by *blaA*, streptogramin inactivation mediated by *vat(F)*, and aminoglycoside inactivation mediated by *ant(3″)-Ia* and *aph(3′)-Ia*. These findings indicate that enzymatic inactivation is a common AMR mechanism among the examined *Y. enterocolitica* isolates, and additional acquired resistance determinants occur only sporadically.

Collectively, the AMR gene profiles of these isolates were characterized by the ubiquitous presence of *blaA* and *vat(F)*, with limited detection of additional aminoglycoside resistance genes. This pattern suggests largely conserved resistance determinants with minimal acquisition of supplementary AMR genes.

#### 3.5.3. Plasmid Replicons

Plasmid replicon analysis revealed that at least one plasmid replicon was detected in 44.0% (40/91) of the isolates (Figure 4). Among the replicon-positive isolates, multiple replicon types were identified at varying frequencies. The most prevalent replicon was Col(pHAD28), detected in 42.5% of replicon-positive isolates, followed by Col440II (20.0%), Col(Ye4449) (17.5%), IncN (15.0%), pYE854, and Col(CriePir75) (10.0% each). Other replicon types were detected at lower frequencies, including ColE10, ColRNAI, and ColpVC (each 7.5%) as well as IncN3 and IncX7 (each 5.0%). Several additional replicons were observed as single occurrences (2.5% each), including IncFII(p14), IncFII(pAR0022), IncFII(pCRY), IncX5, pENTAS02, pIP31758(p153), and pIP31758(p59).

None of the 91 isolates carried the classical pYV virulence plasmid, which is typically associated with pathogenic strains of *Y. enterocolitica*. Instead, the plasmid replicons detected were predominantly Col- and Inc-type replicons, which occurred at relatively low-to-moderate frequencies. These findings indicate that the plasmid replicon profiles among the examined *Y. enterocolitica* isolates are diverse but do not appear to be dominated by the classical virulence plasmid. The detection of multiple replicon types indicates diversity in plasmid-associated sequences within this population. The biological roles of these plasmid-associated sequences require further investigation.

## 4. Discussion

This study provides the first comprehensive genomic characterization of *Y. enterocolitica* isolates recovered from animal-source foods in South Korea. All 91 isolates were predicted as biotype 1A (sub-biotype 1Aa), a lineage traditionally considered nonpathogenic. However, phylogenomic and sequence-typing analyses revealed substantial genetic heterogeneity, comprising 27 STs and 43 CTs.

The partial overlap of cgMLST profiles and the detection of shared cgMLST types across chicken, pork, beef, and duck indicate that genetically related *Y. enterocolitica* lineages were present in multiple animal-source food categories. Similar population structures have been reported in previous studies from Europe and China, where biotype 1A predominated among food-derived isolates and exhibited high ST diversity [22,23]. Nevertheless, the dominant STs vary by region. For example, a study of 158 *Y. enterocolitica* isolates from England identified ST18 as the most prevalent lineage, whereas ST134 was detected only sporadically [23]. Conversely, ST134 was the predominant lineage in this study, accounting for 25.3% of all isolates, indicating regional variation in the distribution of biotype 1A lineages [23]. Collectively, these findings indicate that *Y. enterocolitica* biotype 1A isolates in South Korea represent a genetically heterogeneous population distributed across diverse animal-source food categories.

The occurrence of shared CTs and zero allelic differences across shared assigned loci among isolates recovered from different animal-source food categories indicates genomic similarity within the examined collection. However, because temporal, facility-level, producer-level, and processing-chain metadata were unavailable, epidemiological linkage or cross-contamination could not be evaluated from the present data. Future studies incorporating such metadata would help clarify whether the observed genomic similarities reflect epidemiological linkage or possible cross-contamination along the food production chain.

Pan-genome analysis further revealed substantial genomic variability among the isolates. Of the 11,230 identified gene clusters, only a relatively small proportion constituted the core genome, whereas accessory and unique genes comprised the majority of the pan-genome, suggesting an open pan-genome structure. COG-based functional classification showed that accessory and unique gene clusters were assigned to predicted categories associated with metabolism, defense mechanisms, and stress responses. Further functional studies are required to determine whether these gene clusters contribute to environmental persistence, fitness, or host-associated adaptation. Overall, the phylogenomic and pan-genomic analyses indicate that *Y. enterocolitica* isolates from animal-source foods represent a genetically diverse population characterized by a conserved core genome and an extensive accessory gene repertoire.

Although the canonical virulence plasmid pYV was absent, all isolates retained several chromosomal virulence-associated genes involved in adhesion (*yapE* and *psaC*), invasion (*inv* and *pla*), secretion systems, and enterotoxicity (*ystB*). However, the detection of these genes alone does not demonstrate their expression, protein functionality, or phenotypic contribution. The absence of pYV, together with the widespread detection of *ystB* and other virulence-associated genes, accords with the genomic background previously described for biotype 1A isolates [15,24]. However, phenotypic biotyping and classical biochemical tests were not performed; therefore, the biotype and sub-biotype assignments should be interpreted as genome-based predictions. Additional phenotypic and functional studies are required to determine the biological contributions of these genes.

AMR profiles were characterized by the universal presence of *blaA* and *vat(F)*, indicating a conserved resistance-associated genetic background within the examined *Y. enterocolitica* population. Similarly, WGS-based studies from Brazil and China reported the widespread occurrence of *blaA* and *vat(F)* among *Y. enterocolitica* isolates [20,22]. The low occurrence of additional acquired AMR determinants may reflect differences in isolate sources, antimicrobial exposure, and the distribution of circulating lineages among studies. Resistance to β-lactam antibiotics is commonly reported in *Y. enterocolitica*, and many strains harbor chromosomal β-lactamase genes, including *blaA* [25]. Additionally, *vat* genes encode streptogramin A acetyltransferases that mediate antibiotic inactivation, and streptogramins, such as virginiamycin, have historically been used in livestock production systems [26,27]. Consequently, the widespread occurrence of *blaA* and *vat(F)* among these animal-source food isolates may reflect historical or ongoing antimicrobial selective pressures in food-animal production environments [28]. However, because *blaA* and *vat(F)* were detected in all isolates, their presence alone is insufficient to distinguish whether they primarily represent intrinsic resistance or resistance profiles influenced by antimicrobial selective pressure. In contrast, acquired AMR genes were detected in only two MDR isolates. Specifically, isolate MFDS1030540 carried *aph(3′)-Ia* and MFDS1030554 carried *ant(3″)-Ia*. These findings suggest that the acquisition of additional AMR determinants was relatively uncommon within the analyzed isolates and was mainly restricted to aminoglycoside resistance-associated genes. As phenotypic antimicrobial susceptibility testing was not performed, the present findings describe genotypic AMR profiles, and further testing would be required to determine their correspondence with phenotypic resistance.

Plasmid replicon analysis showed that at least one plasmid replicon was detected in 44.0% of the isolates. Among these isolates, a subset harbored the pYE854 replicon, previously reported in pathogenic *Y. enterocolitica* isolates [19]. The predominant plasmid groups consisted of Col- and Inc-type replicons, which are generally associated with small mobilizable plasmids or plasmid maintenance functions, rather than classical virulence plasmids [29,30]. The detection of multiple replicon types indicates diversity in plasmid-associated sequences within the examined population. The biological roles of these sequences require further investigation.

This study focused on food-derived isolates collected from poultry and red meat in South Korea during 2024 and did not include clinical isolates. Because the numbers of isolates differed among food categories, particularly the small number of duck isolates, source-related patterns were interpreted descriptively. Pathogenicity and antimicrobial resistance were assessed based on genomic characteristics rather than phenotypic testing. Future studies incorporating clinical isolates, phenotypic analyses, and broader geographic and temporal sampling would provide a more comprehensive understanding of the genomic characteristics of *Y. enterocolitica*.

Taken together, the findings indicate that *Y. enterocolitica* isolates recovered from animal-source foods in South Korea constitute a genetically diverse population characterized by conserved chromosomal virulence-associated genes, intrinsic AMR determinants, and diverse plasmid replicons. From a food safety perspective, continued genomic surveillance of *Y. enterocolitica* in animal-source foods and food-processing environments may help track genetic variation, monitor AMR and plasmid profiles, and improve understanding of contamination dynamics.

## 5. Conclusions

In this study, the genomic features of *Y. enterocolitica* isolates recovered from animal-source foods in South Korea in 2024 were characterized. WGS indicated that all isolates were predicted as biotype 1A while exhibiting substantial genomic heterogeneity, an open pan-genome, and a conserved chromosomal backbone. Although multiple chromosomal virulence-associated loci were detected, their presence alone does not demonstrate functional virulence or increased foodborne risk. Because no gene-expression, cell-based, or animal-model experiments were performed, the pathogenic significance of these loci remains to be determined. The detection of diverse Col- and Inc-type plasmid replicons further indicates the presence of accessory plasmid elements within the examined population. Overall, these findings indicate that the examined isolates represent a genetically diverse biotype 1A population. As the isolates analyzed in this study were obtained from animal-source foods collected in 2024, further studies incorporating isolates from broader sources, geographic regions, and time periods, together with phenotypic analyses, would help improve the understanding of *Y. enterocolitica* in South Korea. From a food safety perspective, continued genomic surveillance of *Y. enterocolitica* in animal-source foods and processing environments may support the monitoring of genetic variation, plasmid replicon profiles, and the distribution of virulence- and antimicrobial resistance-associated genes. The present findings provide a genomic baseline for such surveillance and for future genotype–phenotype investigations of biotype 1A *Y. enterocolitica*.

## Figures and Tables

**Table 1:** Overview of isolates and genotyping results.

Category	Description
Isolates (country)	91 (South Korea)
Sources, n (%)	Chicken 32 (35.2), Pork 30 (33.0), Beef 24 (26.4), Duck 5 (5.5)
Regions, n (%)	Gyeonggi 7 (7.7), Gangwon 1 (1.1), Chungcheong 22 (24.2), Jeolla 52 (57.1), Gyeongsang 9 (9.9)
Biotype/Sub-biotype	1A/1Aa
Sequence types (STs)	27 STs; 6 unassigned
Core-genome types (CTs)	43 CTs
Major ST (n) (n ≥ 2)	ST134 (23), ST179 (12), ST954 (5), ST150 (6), ST246 (6), ST248 (4), ST470 (3), ST484 (3), ST568 (3), ST200 (2), ST1125 (2)
Major CT (n) (n ≥ 2)	CT3053 (16), CT2661 (5), CT2483 (4), CT2567 (4), CT2676 (4), CT531 (3), CT1420 (3), CT1548 (3), CT2534 (3), CT2546 (3), CT3096 (3), CT480 (2), CT486 (2), CT488 (2), CT542 (2), CT1398 (2), CT2316 (2), CT2668 (2), CT3063 (2)

## Data Availability

The sequence data generated in this study have been deposited in publicly accessible NCBI repositories, including GenBank, under the accession numbers provided in Table S1.

## Supplementary Materials

The following supporting information can be downloaded at: https://www.mdpi.com/article/10.3390/pathogens15080821/s1, Table S1: Metadata for 91 *Yersinia enterocolitica* strains analyzed in this study; Table S2: Genome quality metrics for the 91 *Yersinia enterocolitica* isolates analyzed in this study; Table S3: Reference *Yersinia enterocolitica* genomes used for biotype and sub-biotype classification; Table S4: Isolate-level cgMLST locus assignment and zero-distance group membership among 91 *Yersinia enterocolitica* isolates; Table S5: Proportions of COG categories detected in the core, accessory, and unique genomes; Table S6: Virulence genes detected in *Yersinia enterocolitica* isolates; Table S7: Antimicrobial resistance genes detected in *Yersinia enterocolitica* isolates.

## Abbreviations

The following abbreviations are used in this manuscript:

AMR	Antimicrobial resistance
cgMLST	core-genome multilocus sequence typing
COG	Clusters of Orthologous Groups
CT	core-genome type
MLST	multilocus sequence typing
ST	sequence type
VF	virulence factor
WGS	whole-genome sequencing
